# Bringing Outdoor Therapies Into Mainstream Mental Health

**DOI:** 10.3389/fpubh.2018.00119

**Published:** 2018-05-03

**Authors:** Ralf C. Buckley, Paula Brough, Diane Westaway

**Affiliations:** ^1^Griffith University, Gold Coast, QLD, Australia; ^2^Coastrek, Sydney, NSW, Australia

**Keywords:** nature, adventure, therapy, outdoor, diagnosis

## Introduction

Several recent reviews have concluded that, while certainly not a panacea, exposure to nature and outdoor activities can improve mental health for at least some symptoms, causes, patients, and circumstances (1–6). They are particularly relevant for the psychological components of chronic disease syndrome, namely depression and dementia (7–9). Outdoor exercise plays a role, in addition to outdoor environments (10–12), but nature-based outdoor activities yield benefits additional to those of exercise alone (13–17). Outdoor therapies can also assist in overcoming some types of chronic pain (18). They are valuable at all ages, from children (19, 20) to seniors (21, 22), and for those with both minor and severe clinical symptoms (23). All of these have substantial economic implications (6, 24, 25).

Currently, however, outdoor therapies have limited deployment, especially in wealthy urbanized nations where they are most valuable. Government health agencies, and private health insurers, run educational and marketing campaigns, but these are for voluntary patient-funded activities. There are various school and youth adventure education programs (19, 20), but these are preventive rather than therapeutic. A few countries have trialed so-called green prescription programs (26), but too small and short to be effective. Some privately run programs may be more successful (27), but they are targeted to specific market sectors, unconnected with mainstream health care. None of these yet provide for routine diagnosis and prescription of outdoor therapies, for patients who present themselves at their general practitioners with mental health concerns. Here, therefore, we consider what additional research may be required to achieve this. There are both social and technical aspects.

The key social obstacle is that outdoor therapies are not yet perceived as mainstream medicine. Even though the practical delivery of outdoor therapies is very similar to widely prescribed physiotherapies and psychotherapies, outdoor therapies are not yet available through publicly or insurance-funded medical diagnosis, prescription, and providers. They are offered principally by private providers, who are forced to adopt business and marketing models more closely aligned to discretionary activities such as the fashion and beauty industry. Historically, similar social obstacles were also faced, and overcome, by many other components of modern health-care systems. It takes time, institutional change, and technical information for them to become routine (1, 6).

Here, we focus on the technical obstacles and the research required to overcome them. Recent reviews agree that knowledge of the therapeutic links between nature exposure and mental health is currently only at proof-of-concept stage, and research is now required to elucidate dose–duration–response relationships (1, 4, 6). We endorse this view and propose two additional areas of research. The first additional area is to differentiate (a) patient symptoms and personality traits and (b) characteristics of therapies, to prescribe specific therapies that match particular patients and conditions. The second is to test the social levers needed to persuade individual patients to adopt and follow through with courses of outdoor therapies once prescribed.

## Characteristics of Patients and Therapies

Research to date has shown that nature exposure can provide a wide range of mental health benefits, related to attention and cognition, memory, stress and anxiety, sleep, emotional stability, and self-perceived welfare or quality of life (1–9, 13, 15–17). As yet, however, there have been no systematic comparisons, cross-testing different outdoor therapies for different mental health conditions. Individuals differ greatly in their psychological and physical capabilities and interests, for different outdoor activities. Some individuals may not be keen to try any outdoor therapies at all. Some patients and conditions may not respond to any outdoor therapies. For those patients and conditions that do respond, different types and intensities of outdoor therapies may prove more effective for different individuals and mental health conditions.

We propose, therefore, that we need to consider these differences explicitly as we establish data on dose, duration, and response, so as to generate a portfolio or menu of outdoor therapies that can be matched to individual patients. Quantifying any individual’s mental health, so as to measure their responses to outdoor therapies, requires a suite of parameters. We can differentiate patients on the basis of symptoms, personality traits or types, capabilities, and interests. These are analogous to factors such as patient body weight, allergies, and drug sensitivities in the use of pharmaceutical treatments, and are equally important. To take just one example, some individuals have sensation-seeking personalities (28), whereas others do not.

Previous research has included many types and intensities of nature exposure, ranging from views from a window (29) to adventure sports involving skill, thrill, and risk (30–33). We can differentiate therapies on the basis of: duration, repetition, and frequency; features of the natural environments concerned; patient activity, including type, degree of physical exercise, and degree of potential risk; and emotional components, such as thrill, fear, or joy (30–33).

Some of these correspond to dose and treatment regime in pharmaceutical therapies, whereas others are analogous to active agents. The former include: the length of each individual period spent outdoors; the time of day when it occurs; the number of occasions per day, week, month, or year; and the overall duration of the treatment regime. The latter include: the ecological, esthetic, and social characteristics of the natural setting where the outdoor activity takes place (34–36); and the type and characteristics of the activity itself (32, 33). Activity characteristics include: physical exercise; strength and skill; risk and thrill; social interactions involved; instructor-led or self-paced; equipment used and safety procedures followed; emotional setting and consequences; and social perception of the activity among the patients’ friends and families, peers, and the general public.

## Diagnosis, Design, and Implementation

Diagnostic tests, questions, and observations are needed to select, design, and prescribe specific outdoor therapies for individual patients. Since research to date is neither systematic nor comprehensive, an adaptive learning approach will be needed. This is acceptable, since the risks are low. Outdoor therapies involve multiple small doses over an extended period of treatment. Treatment regimes can easily be adjusted if adverse effects are detected, or if the dose proves too small to be effective. The primary positive therapeutic effects of outdoor therapies can be detected by individual patients and described to prescribing practitioners, during the course of the therapy. This contrasts with many other types of therapy, where the patient may only be able to detect negative side effects. If a practitioner prescribes an outdoor therapy regime that is too powerful for a particular patient, analogous to exceeding drug tolerance, then the patient simply will not have the physical capability to perform it.

As more people adopt organized, well-defined, and regular outdoor therapies, this will provide opportunities to conduct large-scale longitudinal studies, evaluating outcomes for individuals with different personalities and prior mental health conditions. This applies whether those organized programs are prescribed or self-adopted (27). Participants could provide individual information to an anonymized central repository, in return for comparative information about their place in an overall population. This would gradually establish a data set for multivariate analysis, to identify the most effective treatments for patients with different symptoms and personality traits. Alternatively, meta-analyses of data sets published with more restricted studies may yield similar results.

Meanwhile, one option is to create a menu of outdoor therapies as a basis for discussion between patient and practitioner, to allow for a voluntary selection. There are a number of considerations. The first is safety: what can the patient be expected to do, without putting themselves at risk? For example, a person with cognitive impairment might not be able to navigate outdoors without assistance. Second, what is the patient’s physical skill and capability? For example, a person who is old, unfit, or overweight might be unable to complete a long outdoor hike, even at slow speed under easy conditions. Third, what are the patient’s prior skills? For example, a person might have prior expertise in photography, plant or animal identification, or a range of adventure recreation activities. Fourth, what does the patient know and enjoy about outdoor nature? In particular, do they prefer passive contemplation and observation, or active exercise?

The menu need not provide a perfect match between patient and therapy, because outdoor therapies are easily adjustable, with low risk of adverse effects. The limiting factor is neither diagnosis nor detailed design, but implementation: persuading patients to commence and persevere. We suggest that simply providing people with information about individual benefits is ineffective. More successful strategies seem to be those that package outdoor therapies as purchasable products. The most effective bundles include multiple social levers operating in parallel (27). Two levers are particularly powerful. The first is *social justification*, to allow individuals to enjoy spending time and funds on personal outdoor nature-based activities, without criticism from family or friends. The second is *mutual peer support*, to prevent relapses under competing pressures.

Here, we suggest two more: an *immediate emotional benefit* and an *irrecoverable investment*. If participants feel happier after spending time in outdoor nature, they will find ways to do so more often. And if they have paid in advance for an experience or program, they are less likely to cancel. Commercial tourism products such as wildlife safaris, for example, satisfy both these criteria. Products offered by some enterprises also contribute directly to the well-being of impoverished communities and the conservation of threatened plant and animal species, adding the social altruism lever identified in previous research (27). In contrast to past public health initiatives relying on untargeted education, therefore, we suggest that designing and marketing highly targeted commercial programs may prove more effective. In slogan form: sell, do not tell.

## Author Contributions

All authors contributed to research. RB wrote final text.

## Conflict of Interest Statement

The authors declare that the research was conducted in the absence of any commercial or financial relationships that could be construed as a potential conflict of interest. The reviewer STE and the handling Editor declared their shared affiliation.
